# Knowledge of Anticoagulation Among Saudi Patients With Atrial Fibrillation: A Cross-Sectional Study

**DOI:** 10.7759/cureus.19237

**Published:** 2021-11-03

**Authors:** Hamdan N Alajami, Sulaiman A Alshammari, Dalal S Al-Dossari, Abdullah N Alajmi, Alanoud S Alsaikhan, Maha S Alessa, Haifa S Alessa, Saud Khalaf Alhothaly, Mohammed I Alnami, Tesfay M Atey, Rashid H Alnajrani, Sheraz Ali

**Affiliations:** 1 Pharmaceutical Care Services, King Saud Medical City, Ministry of Health, Riyadh, SAU; 2 Family and Community Medicine, King Saud University, College of Medicine, Riyadh, SAU; 3 Pharmaceutical Care Division, King Faisal Specialist Hospital and Research Centre, Riyadh, SAU; 4 College of Dentistry, King Saud University, Riyadh, SAU; 5 School of Pharmacy and Pharmacology, University of Tasmania, Hobart, AUS; 6 School of Pharmacy, Mekelle University, Tigray, ETH

**Keywords:** atrial fibrillation, patient knowledge, noacs, warfarin therapy, direct oral anticoagulation

## Abstract

Background

Knowledge about oral anticoagulant treatment can impact treatment outcomes in patients with atrial fibrillation. However, evidence is scarce regarding the knowledge of oral anticoagulants among Saudi patients with atrial fibrillation. Hence, this study aimed to assess the level of anticoagulation knowledge among patients with atrial fibrillation taking oral anticoagulants.

Methodology

A survey using a cross-sectional study design was conducted among patients with a confirmed diagnosis of atrial fibrillation in a tertiary care setting. The Oral Anticoagulation Knowledge Tool (AKT), a 33-item, self-administered questionnaire, was used to assess the knowledge of anticoagulation.

Results

A total of 290 patients with a median age of 67 years participated in the survey. More than half of those surveyed (56.2%) were females. Overall, 195 (67.2%) patients had an overall adequate anticoagulation knowledge. The median knowledge score of participants on warfarin was significantly higher than those on direct-acting oral anticoagulants (*p* < 0.001). Only age was found to be a predictor of AKT. Increasing age was associated with fewer odds of adequate AKT. For every one-year increase in age, the knowledge score decreased by 0.08 (95% confidence interval: -0.13 to -0.04).

Conclusions

This study found significant knowledge gaps among Saudi patients with atrial fibrillation taking oral anticoagulants. Advancing age was inversely associated with oral anticoagulation knowledge.

## Introduction

Atrial fibrillation is the most common type of arrhythmia in medical practice [1]. Patients with atrial fibrillation have a five times greater risk of developing ischemic stroke [2]. In modern society, the use of oral anticoagulants is common due to their use in numerous indications, ranging from atrial fibrillation to mechanical heart valves [3,4]. However, several factors can limit the optimal use of oral anticoagulants [5]. Oral anticoagulants are classified into the following groups: direct-acting oral anticoagulants (DOACs), such as dabigatran, apixaban, rivaroxaban, and edoxaban, and vitamin K antagonists (VKAs) such as warfarin [6,7].

The prevalence of atrial fibrillation remains unknown in the Kingdom of Saudi Arabia (KSA); however, the findings of the first registry study of Saudi patients with atrial fibrillation revealed a higher use of oral anticoagulants (75%) [8]. In other countries, the use of oral anticoagulants in patients with atrial fibrillation ranges from 32% to 64% [9].

Evidence indicates that collaborative decision-making with patients is crucial in the management of atrial fibrillation [10]. Similarly, patients with atrial fibrillation need to be appropriately informed about their disease and the use of oral anticoagulants. Oral anticoagulants are risk-prone drugs, and complex patient understanding is vital for promoting the rational use of oral anticoagulants [11]. Moreover, knowledge of oral anticoagulant therapy can affect treatment outcomes in patients with atrial fibrillation [12]. Patients with better knowledge of the risk and benefit of oral anticoagulant therapy exhibit better adherence than those with inadequate knowledge [5]. Moreover, patients’ awareness of their disease and oral anticoagulant therapy have been described to positively influence anticoagulation control [12,13]. Factors that improve outcomes in patients taking oral anticoagulants include increased adherence to dosing schedules, knowledge of international normalized ratio (INR), regulation of diet to avoid interactions, and monitoring the side effects of oral anticoagulants [14].

Oral anticoagulants remain a high-risk treatment approach despite numerous advantages of DOACs over warfarin [1]. It is crucial to ascertain the adequacy of anticoagulation knowledge in patients who are prescribed oral anticoagulants and to address any misconceptions. In the KSA, evidence is scarce regarding the knowledge of oral anticoagulants among patients with atrial fibrillation as previous reports [15,16] focused on assessing the knowledge of healthcare professionals toward oral anticoagulants. Therefore, in this study, we aimed to assess the level of anticoagulation knowledge among patients with atrial fibrillation taking oral anticoagulants. The findings of this study will help identify knowledge deficits and help develop customized education for patients with atrial fibrillation.

## Materials and methods

This cross-sectional study was conducted from August to September 2021 at the King Saud University Medical City, a tertiary care setting in Riyadh, KSA. This setting serves a broad range of patients drawn from a large population, several of whom present with complex clinical conditions.

Patients aged 18 years and over, with a confirmed diagnosis of atrial fibrillation, who were using oral anticoagulants for at least three months were included in the study. Pediatric patients, patients without atrial fibrillation, healthcare professionals, and those unwilling to participate were excluded. The Oral Anticoagulation Knowledge Tool (AKT), a 33-item, self-administered questionnaire for assessing the knowledge of anticoagulation, was distributed among patients with atrial fibrillation at the King Saud University Medical City. This questionnaire was adapted from a previous study which showed acceptable validity and reliability [17]. The questionnaire was used to collect demographic data, assess the patient’s knowledge of oral anticoagulants, and information about patients on warfarin therapy. For each question, 1 point was awarded for each correct response and 0 for each wrong response, except for questions 18 and 19 in section 2. In these questions, 1 point was given for each correct option out of 3. Sections 2 and 3 were scored out of 24 and 10, respectively. Section 3 was only provided to those patients on warfarin therapy. Final scores were presented as a percentage of correct responses for all participants in the study. A cut-off of >50% was considered as an adequate knowledge score.

All participants were briefed about the goal of the study and provided a study information sheet. A waiver of written informed consent was granted by the ethics committee. This research project was commenced after the approval of the Institutional Review Board of King Saud University Medical City (Reference number: 21/0691/IRB). This study was conducted according to the principles of the Declaration of Helsinki.

In a previous study, 74% of respondents had adequate knowledge regarding oral anticoagulants [18]. Given this proportion, a 95% confidence level, and a 5% margin of error, a sample size of 292 was estimated using an online calculator [19]. Testing for normality of continuous variables was performed using the Shapiro-Wilk test. Baseline characteristics of the participants were reported using percentages and medians (±interquartile range [IQR]). Mann-Whitney U test (two groups) or Kruskal-Wallis test (more than two groups) was applied to compare the non-parametric data. A binary logistic regression was conducted to identify factors associated with adequate AKT. As a subanalysis, linear regression was also conducted to determine the presence of a relationship between the mean AKT score and age. All analyses were declared significant at p-values of <0.05. Data were analyzed using R® (version 4.0.5).

## Results

Table 1 presents the demographic characteristics of the 290 study participants and the median knowledge scores. The median age was 67 years (IQR: 60-73.75). More than half of those surveyed (56.2%) were females. One in two participants had no formal education and were either unemployed or retired. As many as one-fourth of the respondents (24.5%) had a monthly income of less than 5,000 Saudi Riyals. Nearly two-thirds of the respondents (64.1%) claimed that they were taking oral anticoagulants for more than two years.

**Table 1:** Demographic characteristics. *Bachelor and postgraduate degree. IQR: interquartile range

Demographic characteristics	n (%)	Median score (IQR)			
		Knowledge of anticoagulant	P-value	Knowledge specific to warfarin	P-value
Age			0.004		0.527
Median (IQR) in years	67 (60–73.75)				
Less than 65 years	121 (41.7)	19 (16–20)		0 (0–4)	
65 years and over	169 (58.3)	18 (14–19)		0 (0–2)	
Sex			0.306		0.349
Female	163 (56.2)	18 (14–20)		0 (0–3)	
Male	127 (43.8)	18 (15–20)		0 (0–2)	
Highest level of education			0.060		0.116
No formal education	145 (50.0)	18 (14–19)		0 (0–3)	
High school or equivalent	89 (30.7)	19 (16–20)		0 (0–1)	
Technical or vocational	7 (2.4)	19 (17–21)		0 (0–0)	
College	14 (4.8)	18.5 (16.2–19.8)		1 (0–3.7)	
University degree*	35 (12.1)	16 (14.5–19)		0 (0–4)	
Occupation			0.900		0.115
Full-time work	15 (5.2)	18 (12–20)		3 (0–4.5)	
Housewife	124 (42.8)	18 (14–20)		0 (0–2)	
Part-time work	3 (1.0)	19 (11–19)		5 (2.5–5.5)	
Unemployed/retired	148 (51.0)	18 (15–20)		0 (0–2)	
Monthly income by Saudi Riyals			0.864		0.862
Less than 5,000	71 (24.5)	18 (15.5–20)		0 (0–2)	
5,001–10,000	24 (8.3)	19 (16.8–20)		0 (0–2.5)	
10,001–15,000	8 (2.8)	16.5 (15.5–18)		0 (0–4.7)	
More than 15,000	2 (0.7)	17.5 (17.2–17.8)		3 (1.5–4.5)	
Prefer not to say	185 (63.8)	18 (15–20)		0 (0–2)	
Duration of oral anticoagulants			0.372		0.054
Not on any medication	14 (4.8)	17 (12.5–18)		0 (0–0)	
Less than 3 months	13 (4.5)	18 (16–19)		0 (0–0)	
3–12 months	43 (14.8)	18 (14.5–19)		0 (0–0)	
1–2 years	34 (11.7)	19 (15–20)		0 (0–4)	
More than 2 years	186 (64.1)	18 (15–20)		0 (0–3)	

No statistical evidence was found for associations between demographic characteristics and the median scores of knowledge specific to warfarin. There was also no evidence that sex, occupation, monthly income, or duration of anticoagulant therapy impacted the median scores of AKT. By contrast, respondents aged less than 65 years had a relatively higher median AKT score compared to those 65 years and older (p = 0.004).

Responses to the knowledge tests of oral anticoagulants and knowledge specific to warfarin therapy are detailed in Table 2. A total of 195 (67.2%) patients had an overall adequate anticoagulation knowledge. One in five of those surveyed (19.0%) claimed that they did not know their medicine’s name. The majority of those who responded indicated the importance of taking the medicines at the same time each day (89.0%), not doubling the next dose if they miss a dose (87.6%), not stopping the medicine even if they feel better (89.0%), not taking more than the prescribed dose (89.3%), informing clinicians before undergoing surgery or procedure (94.1%), and informing all clinicians that they are taking the medicine (96.6%). However, more than half of the respondents (54.8%) did not know exactly why they were taking the oral anticoagulants.

**Table 2:** Knowledge of oral anticoagulants and knowledge specific to warfarin therapy. DVT: deep vein thrombosis; BP: blood pressure; INR: international normalized ratio

Knowledge of oral anticoagulants	Response, n (%)
What is the name of your anticoagulant medicine?	
Apixaban	2 (0.7)
Dabigatran	14 (4.8)
Rivaroxaban	105 (36.2)
Warfarin	114 (39.3)
Do not know the name	55 (19.0)
Why has your doctor prescribed you this medicine?	
Arrhythmias	108 (37.2)
Blood-thinning	64 (22.1)
Cardiac issue/chest pain	33 (11.4)
DVT	45 (15.5)
Prosthetic valve	21 (7.2)
Others	4 (1.4)
Do not know	15 (5.2)
How does this medicine work in your body?	
Lowers BP	5 (1.7)
Prevents blood from clotting	184 (63.4)
Lowers heart rate	19 (6.5)
Do not know	82 (28.3)
How many times a day do you need to take this medicine?	
Once	237 (81.7)
Twice	6 (2.1)
Thrice	41 (14.1)
Do not know	6 (2.1)
For how long do you need to take this medicine?	
3 months	12 (4.1)
6 months	14 (4.8)
1 year	3 (1.0)
Lifelong	182 (62.8)
Do not know	79 (27.2)
Why is it important to take this medicine exactly as your doctor has told you?	
Too much of this can cause bleeding	94 (32.4)
It interacts with food, so changing the dose/timing can be hazardous	37 (12.8)
Do not know	159 (54.8)
Is it important to take this medicine at the same time each day?	
Yes	258 (89.0)
No	17 (5.9)
Not sure	14 (4.8)
Is it okay to double the next dose of this medicine if you miss a dose?	
Yes	12 (4.1)
No	254 (87.6)
Not sure	24 (8.3)
Is it possible that skipping one dose of this medicine could worsen your condition?	
Yes	39 (13.4)
No	118 (40.7)
Not sure	133 (45.9)
Is it appropriate to stop taking this medicine once you feel better?	
Yes	258 (89.0)
No	19 (6.5)
Not sure	13 (4.5)
Is it safe to take anti-inflammatory medicines like ibuprofen (Nurofen^®^ or Advil^®^) while you are taking this medicine?	
Yes	53 (18.3)
No	128 (44.1)
Not sure	109 (37.6)
Is it safe to take vitamin supplements and herbal medicines with this medicine without consulting your doctor?	
Yes	50 (17.2)
No	202 (69.7)
Not sure	38 (13.1)
Is there any benefit in taking more of this medicine than your doctor has told you to take?	
Yes	259 (89.3)
No	14 (4.8)
Not sure	17 (5.9)
Will drinking too much alcohol increase the risk of side effects with this medicine?	
Yes	130 (44.8)
No	73 (25.2)
Not sure	87 (30.0)
Would you inform a surgeon, dentist or other health professional that you are taking this medicine before undergoing surgery or a procedure?	
Yes	273 (94.1)
No	10 (3.5)
Not sure	7 (2.4)
Is it important that all the health care practitioners you see know that you are taking this medicine?	
Yes	280 (96.6)
No	2 (0.7)
Not sure	8 (2.8)
What is the most important side effect of this medicine?	
Bleeding	132 (45.5)
Others	28 (9.7)
Do not know	130 (44.8)
Three signs of side effects that you should watch out for while taking this medicine are	
Bleeding gums	152 (52.4)
Prolonged nosebleeds	107 (36.9)
Severe bruising	150 (51.7)
Blood in urine	105 (36.2)
Insomnia	38 (13.1)
Loss of appetite	34 (11.7)
Do not know	94 (32.4)
Three things you can do to reduce your risk of side effects are:	
Monitor INR regularly	74 (25.5)
Monitor side effects	129 (44.5)
Sleeping on time	117 (40.3)
Eating less food	93 (32.1)
Avoid things that could cause cuts/injuries	84 (29.0)
Proper dosing	95 (32.8)
Do not know	92 (31.7)
What is the best step to take if you accidentally take too much of this medicine?	
Skip the next dose	28 (9.7)
Consult my doctor	170 (58.6)
Be alert for signs of side effects	47 (16.2)
Do not know	45 (15.5)
Knowledge specific to warfarin therapy (n = 120)	Response, n (%)
What is your target INR range?	
<1.0	1 (0.3)
1.0 to 1.9	3 (1.0)
2.0 to 3.0	54 (18.6)
>3.0	7 (2.4)
Do not know	54 (18.6)
What was your last INR reading?	
<1.0	7 (2.4)
1.0 to 1.9	3 (1.0)
2.0 to 3.0	54 (18.6)
>3.0	7 (2.4)
Do not know	54 (18.6)
Are regular INR tests necessary to know how well this medicine is working?	
Yes	85 (29.3)
No	1 (0.3)
Not sure	34 (11.7)
Is an INR value above your target range good for your general wellbeing?	
Yes	5 (1.7)
No	60 (20.7)
Not sure	55 (19.0)
Is it possible for INR values below your target range to be bad for your health?	
Yes	49 (16.9)
No	18 (6.2)
Not sure	53 (18.3)
Is it possible for what you eat to affect your warfarin therapy?	
Yes	79 (27.2)
No	13 (4.5)
Not sure	28 (9.7)
List one vitamin that can significantly affect your anticoagulant therapy	
Vitamin A	4 (1.4)
Vitamin B	0 (0)
Vitamin K	30 (10.3)
Do not know	81 (27.9)
Others	6 (2.1)
Total knowledge score	
Adequate	195 (67.2%)
Inadequate	95 (32.8%)

There were numerous important differences between participants taking DOACs and warfarin (Table 3). The median knowledge score of participants on warfarin was significantly higher than those on DOACs (p < 0.001). Participants taking warfarin had a significantly higher score on questions regarding the mechanism of action and the most important side effects than those taking DOACs.

**Table 3:** Items with considerable differences between participants taking direct oral anticoagulants and warfarin. DOACs: direct oral anticoagulants; IQR: interquartile range

Item	DOACs (N = 121); n (%)		Warfarin (N = 114); n (%)		P-value
	Incorrect	Correct	Incorrect	Correct	
Why has your doctor prescribed this medicine	6 (5.0)	115 (95.0)	9 (7.9)	105 (92.1)	<0.001
How does this medicine work in your body	68 (56.2)	53 (43.8)	22 (19.3)	92 (80.7)	0.003
Is it okay to double the next dose of this medicine if you missed a dose	10 (8.3)	111 (91.7)	21 (18.4)	93 (81.6)	0.035
Is it safe to take vitamin supplements and herbal medicines with this medicine without consulting your doctor	17 (14.0)	104 (86.0)	35 (30.7)	79 (69.3)	0.003
What is the most important side effect of this medicine	73 (60.3)	48 (39.7)	48 (42.1)	66 (57.9)	0.008
Knowledge score, median (IQR)	17 (15–20)		22 (17–24.8)		<0.001

**Figure 1:**
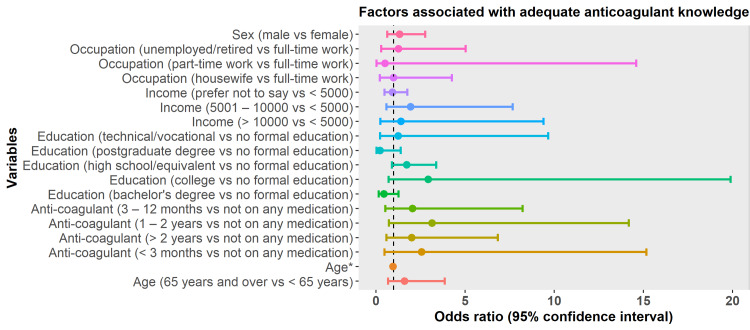
Results of logistic regression analysis for factors associated with adequate anticoagulant knowledge. *Statistically significant at p < 0.05.

**Figure 2:**
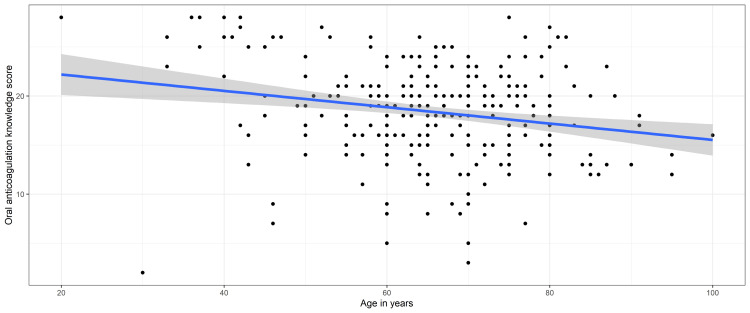
Relation between oral anticoagulation knowledge score and age of the respondents (filled circles). The straight line is the oral anticoagulation knowledge score estimated by linear regression and the gray region depicts the 95% confidence interval for the mean.

The multivariate logistic regression showed that only age was a predictor of AKT. Increasing age was associated with fewer odds of having adequate AKT (odds ratio [OR]: 0.955; 95 confidence interval [CI]: 0.920-0.991). There was no evidence that sex, income, education, occupation, or duration of anticoagulant therapy had an impact on the AKT score (Figure 1). Further analysis using linear regression for the predicted relation between age and oral anticoagulation knowledge score is depicted in Figure 2. There was strong evidence that the age of the respondents had an effect on the oral anticoagulation knowledge score (linear regression; F1,288 = 13.9; p < 0.001). For every one-year increase in age, the knowledge score decreased by 0.08 (95% CI: -0.13 to - -0.04).

## Discussion

This study assessed the knowledge level regarding anticoagulation in Saudi patients with atrial fibrillation and compared the knowledge level between those taking warfarin and DOACs. A total of 195 (67.2%) patients had an overall adequate knowledge of anticoagulation. The median knowledge score of participants on warfarin was significantly higher than for those on DOACs. An implication of this is the possibility of differences in the management of anticoagulants. Unlike vitamin K antagonists (i.e., warfarin), DOACs are mostly self-managed, without a requirement for regular coagulation monitoring, and have a short half-life. Overall, knowledge gaps were noticed in key areas of rational prescribing and self-management, including omitted doses, drug-drug interactions, and acknowledging bleeding as an important side effect of the medicine. This evidence is consistent with those of other reports across several populations in which suboptimal knowledge of oral anticoagulants has been frequently reported [13,18,20,21].

Our findings indicate that the knowledge of oral anticoagulants in older people aged 65 years and above is significantly lower than participants aged less than 65 years. Likewise, age was also a predictor of anticoagulants’ knowledge in regression analysis. The effect of age on the knowledge of oral anticoagulants has been investigated in varied populations, and progressing age has been identified to be inversely correlated with the knowledge of oral anticoagulants in numerous reports [12,20-23]. Nonetheless, the relationship between other demographic characteristics and oral anticoagulants’ knowledge is not fully understood as different studies have reported varied results. Therefore, our result aligns well with the broader scientific literature.

Patients on warfarin had significantly higher levels of knowledge than patients on DOACs. This finding is consistent with the results of the national survey conducted in Australia [21]. There are numerous possible explanations for this finding as participants taking warfarin therapy may have received more intensive medication counseling, particularly if dose modification was required after an INR test. On the contrary, due to the relative simplicity of DOACs, patients on DOAC could have had fewer regular interactions with healthcare professionals. The frequency and the quality of patient-healthcare professional interactions are recognized as a determinant of drug-related knowledge [24]; the frequency of patient-healthcare professional interactions is perceivably more in patients on warfarin compared with DOAC given the requirement for regular INR monitoring.

Participants with adequate knowledge about anticoagulation can better contribute to shared decision-making and self-management of their illness. This study has shown that the knowledge of oral anticoagulants is suboptimal in participants taking DOACs. It is crucial to integrate knowledge assessment within counseling programs and be provided to patients with atrial fibrillation at the commencement of their oral anticoagulant therapy and continually thereafter to explore and address an awareness gap. In the absence of regular coagulation monitoring for DOACs, a similar follow-up session needs to be introduced for DOAC consumers to assess the knowledge of oral anticoagulants and other patient-related outcomes.

In the KSA, further research is required to ascertain reasons for the lower knowledge levels in patients on DOACs, as well as the impact of medication counseling and education intervention on the knowledge score of Saudi patients prescribed oral anticoagulants in general. However, this study has certain limitations. This study was cross-sectional and conducted in a single healthcare institution, and thus cannot inform about causality and the findings cannot be generalized to the entire Saudi population. Therefore, it is prudent to conduct a national survey with a large sample size between Saudi patients with atrial fibrillation taking warfarin and DOACs.

## Conclusions

This study found significant knowledge gaps in Saudi participants with atrial fibrillation taking oral anticoagulants, and these gaps appear to be greater in patients on DOACs. Advancing age was inversely associated with oral anticoagulation knowledge. This evidence may increase awareness of patient-related determinants that can influence treatment outcomes in oral anticoagulation therapy. Future studies can explore the influence of a multidisciplinary education program on anticoagulation knowledge in patients with atrial fibrillation taking warfarin and DOACs.
